# Pre-sleep milk-derived proteins do not impair sleep or recovery in elite female athletes

**DOI:** 10.1080/15502783.2025.2550204

**Published:** 2025-08-29

**Authors:** Kieran G. P. Paterson, Timothy D. Griest, Matthew D. Vukovich, Michael J. Ormsbee

**Affiliations:** a Florida State University, Department of Health, Nutrition, and Food Sciences, Tallahassee, USA; b Florida State University, Institute of Sports Sciences & Medicine, Tallahassee, USA; c South Dakota State University, College of Education and Human Sciences, Vermillion, USA; d University of KwaZulu-Natal, School of Health Sciences, Discipline of Biokinetics, Exercise and Leisure Sciences, Durban, South Africa

**Keywords:** Sleep quality, female athletes, Nutritional strategy, recovery

## Abstract

**Purpose:**

Sleep is essential for recovery in athletes and overall health. While pre-sleep feeding (PSF), defined as consuming calories 2 hours after the last meal and 30 minutes before bed, is theorized to influence sleep and recovery, there is both a paucity of and conflicting data on whether PSF improves or impairs sleep quality and recovery. α-Lactalbumin (a milk-derived protein), in particular, is hypothesized to be a potentially superior PSF option due to its high tryptophan content, yet little data exist regarding its efficacy compared to other strategies.

**Methods:**

This randomized, double-blind, crossover study investigated the effects of four pre-sleep feeding treatments over a 4–8 week period in elite NCAA Division 1 female athletes (*n* = 25). Each participant consumed 40 g of: (1) α-Lactalbumin (ALA); (2) casein protein (CAS); (3) carbohydrate (CHO); or (4) non-caloric placebo (PLA) mixed with water, for three consecutive nights, two hours after last meal, 30 mins before bed. Diets were tracked using food logs. Objective sleep (total sleep time [TST]) and recovery metrics (heart rate variability [HRV] and resting heart rate [RHR]) were collected continuously using a wrist-worn device. Daily morning questionnaires assessed subjective sleep (subjective sleep quality [SSQ] and subjective sleep onset latency [SSOL]) and subjective recovery (SREC). Data were analyzed using repeated measures ANOVA.

**Results:**

Twenty-one (*n* = 21) female athletes completed the study (body mass: 66.89 ± 11.85 kg; body fat% %: 27.28 ± 4.57%; height: 1.69 ± 0.07 m, BMI of 23.28 ± 2.96 kg/m^2^; FFMI of 16.85 ± 1.74 kg/m^2^). No significant differences were observed between the four treatments for any objective sleep metrics (TST: PLA 7.52 ± 1.11 h; CHO 7.62 ± 1.06 h; ALA 7.53 ± 0.89 h; CAS 7.47 ± 1.14 h; *p* = 0.91), subjective sleep metrics (SSQ: PLA 71.31 ± 20.60; CHO 74.88 ± 17.81; ALA 72.92 ± 15.39; CAS 69.12 ± 16.66; *p* = 0.35; SSOL: PLA 16.62 ± 14.08 min; CHO 16.45 ± 12.28 min; ALA 20.21 ± 16.83 min; CAS 18.09 ± 18.71 min; *p* = 0.20), or objective and subjective recovery metrics (RHR: PLA 53.71 ± 6.55 bpm; CHO 54.05 ± 6.66 bpm; ALA 53.54 ± 6.19 bpm; CAS 53.71 ± 5.42 bpm; *p* = 0.99; HRV: PLA 98.24 ± 34.01 ms; CHO 99.14 ± 34.08 ms; ALA 103.91 ± 31.48 ms; CAS 103.05 ± 34.60 ms; *p* = 0.54; SREC: PLA 65.93 ± 20.40; CHO 65.64 ± 19.22; ALA 64.73 ± 17.06; CAS 60.93 ± 19.76; *p* = 0.43).

**Conclusion:**

PSF, specifically a 40 g protein bolus, does not negatively affect objective or subjective sleep quality or recovery in elite female athletes. In addition, α-lactalbumin (43% α-Lactalbumin) does not influence sleep more than other pre-sleep protein options. PSF may provide a valuable opportunity to meet calorie and macronutrient needs, especially protein, essential for supporting recovery and adaptation in this population, without impacting sleep.

